# Southern African HIV Clinicians Society adult antiretroviral therapy guidelines: Update on when to initiate antiretroviral therapy

**DOI:** 10.4102/sajhivmed.v16i1.428

**Published:** 2015-12-03

**Authors:** Graeme Meintjes, John Black, Francesca Conradie, Sipho Dlamini, Gary Maartens, Thandekile C. Manzini, Moeketsi Mathe, Michelle Moorhouse, Yunus Moosa, Jennifer Nash, Catherine Orrell, Francois Venter, Douglas Wilson

**Affiliations:** 1 Department of Medicine and Institute of Infectious Disease and Molecular Medicine, University of Cape Town, South Africa; 2 Department of Medicine, Livingstone Hospital, South Africa; 3 Right to Care and Clinical HIV Research Unit, University of the Witwatersrand, South Africa; 4 Division of Infectious Diseases & HIV Medicine, Department of Medicine, University of Cape Town, South Africa; 5 Division of Clinical Pharmacology, University of Cape Town, South Africa; 6 Department of Infectious Diseases, King Edward VIII Hospital, University of KwaZulu-Natal, South Africa; 7 Private practice, Vereeniging, South Africa; 8 Wits Reproductive Health and HIV Institute, Johannesburg, South Africa; 9 Department of Infectious Diseases, University of KwaZulu-Natal, South Africa; 10 Amathole District Clinical Specialist Team, Eastern Cape, South Africa; 11 Desmond Tutu HIV Foundation, University of Cape Town, South Africa; 12 Department of Internal Medicine, Edendale Hospital, University of KwaZulu-Natal, South Africa

## Abstract

The most recent version of the Southern African HIV Clinicians Society’s adult antiretroviral therapy (ART) guidelines was published in December 2014. In the 27 August 2015 edition of the *New England Journal of Medicine*, two seminal randomised controlled trials that addressed the optimal timing of ART in HIV-infected patients with high CD4 counts were published: Strategic timing of antiretroviral therapy (START) and TEMPRANO ANRS 12136 (Early antiretroviral treatment and/or early isoniazid prophylaxis against tuberculosis in HIV-infected adults). The findings of these two trials were consistent: there was significant individual clinical benefit from starting ART immediately in patients with CD4 counts higher than 500 cells/μL rather than deferring until a certain lower CD4 threshold or clinical indication was met. The findings add to prior evidence showing that ART reduces the risk of onward HIV transmission. Therefore, early ART initiation has the public health benefits of potentially reducing both HIV incidence and morbidity. Given this new and important evidence, the Society took the decision to provide a specific update on the section of the adult ART guidelines relating to when ART should be initiated.

## The 2014 guidelines

In the 2014 version of the Southern African HIV Clinicians Society’s adult antiretroviral therapy guidelines, antiretroviral therapy (ART) was recommended for certain clinical indications (including WHO stage 3 and 4 disease, and other significant morbidities), for HIV-infected partners in serodiscordant relationships, and in all patients with a CD4 < 350 cells/μL. Further, these guidelines advised that if patients had two CD4 counts between 350 and 500 cells/μL, ART should be started if the patient was ready and motivated to start ART. However, in these guidelines it was advised that if a patient’s CD4 count was > 500 cells/μL and the patient did not qualify on clinical grounds, then ART should be deferred. The rationale was that insufficient evidence existed to advise a ‘test-and-treat’ approach to HIV at that time, and it was stated that we awaited the results of the TEMPRANO and Strategic timing of antiretroviral therapy (START) trials.^1^

For patients diagnosed during acute HIV seroconversion, initiation of standard first-line ART was advised, provided that adherence requirements were met. For such patients, it was advised that ART should be continued for at least 3 years, but consideration should be given to continuing lifelong ART.

## The strategic timing of antiretroviral therapy and TEMPRANO trials

Both the START and TEMPRANO trials enrolled patients with high CD4 counts (in START all participants had a study entry CD4 count > 500 cells/μL; in TEMPRANO, 41% had an entry CD4 count > 500 cells/μL). A summary of the trials is presented in Table 1. Both trials involved randomisation to one of two strategies: immediate ART initiation, or to defer ART until the participant was eligible on the basis of CD4 count or clinical criteria. Patients were followed for about 3 years in each trial, and the primary endpoint was a composite endpoint that included AIDS events, serious non-AIDS events and death, with minor differences in specific aspects of the endpoint definition between the two trials.

**TABLE 1:** Summary of design, conduct and findings of the Strategic timing of antiretroviral therapy and TEMPRANO ANRS 12136 (Early antiretroviral treatment and/or early isoniazid prophylaxis against tuberculosis in HIV-infected adults) randomised controlled trials.

Trial	TEMPRANO	START
Countries	Cote d’Ivoire	35 countries (21% of participants enrolled in Africa)
Enrolment years	2008–2012	2009–2013
Number of participants	2056	4685
Inclusion criteria	≥ 18 years old	≥ 18 years old
	HIV-1 (or dual HIV-1 and 2)	ART naive
	CD4 < 800	No history of AIDS
	Not meeting WHO criteria for starting ART at the time (these criteria changed during the course of the trial)	General good health
	-	2 CD4 counts > 500
Comparison arms	*Immediate* ART	*Immediate* ART
	ART *deferred* until WHO criteria for starting ART met (these criteria changed over the course of the trial)	ART *deferred* until CD4 ≤ 350, AIDS diagnosis or other indication for ART (e.g. pregnancy)
Composite primary endpoint	AIDS, non-AIDS cancer, non-AIDS invasive bacterial disease or death	Serious AIDS-related event, serious non-AIDS-related event or death
Duration of follow-up	30 months for each participant	Mean 3.0 years (trial stopped early by DSMB)
Number of primary events	Immediate arm: 64	Immediate arm: 42
	Deferred arm: 111	Deferred arm: 96
Primary endpoint finding	44% reduction with immediate ART (aHR = 0.56, 95% CI = 0.41–0.76)	57% reduction with immediate ART (HR = 0.43, 95% CI = 0.30–0.62)
	Among patients with baseline CD4 ≥ 500, there was also a 44% in primary endpoint (aHR = 0.56, 95% CI = 0.33–0.94)	-
Main contributors to finding	Reduction in AIDS events (50%, mainly TB [50%]) and invasive bacterial disease (61%)	Reduction in AIDS events (72%, including TB [71%]), serious non-AIDS events (29%), cancers (64%) and bacterial infections (62%)
Deaths	Immediate arm: 21	Immediate arm: 12
	Deferred arm: 26	Deferred arm: 21
	Not significant: aHR = 0.60, 95% CI = 0.34–1.09	Not significant: *p* = 0.13
Viral load suppression	Viral load < 100 at 12 months on ART	Viral load < 200 at 12 months on ART
	Immediate arm: 84%	Immediate arm: 98%
	Deferred arm: 80%	Deferred arm: 97%
Adverse events	Overall, the 30-month probability of a Grade 3 or 4 AE did not differ between arms although it was 2.6 times higher in the immediate ART arm for the first 6 months	No difference between arms in terms of grade 4 events and hospitalisations for reasons other than AIDS

Note: In the TEMPRANO trial, there was a separate randomisation of participants to 6 months isoniazid preventive therapy (IPT) versus no IPT.

WHO, World Health Organization; DSMB, Data and Safety Monitoring Board; aHR, adjusted hazard ratio; CI, confidence interval; HR, hazard ratio; AE, adverse event.

Both trials demonstrated a statistically significant, approximate halving of events contributing to the primary endpoint when ART was started immediately. In TEMPRANO, this benefit was largely attributable to reductions in incident TB and invasive bacterial diseases (particularly pneumonia). In START, the benefit was related to a decrease in AIDS-related events (including TB) and serious non-AIDS events (including cancer). The relative reduction in the rate of primary endpoint events was greater in START (57% reduction compared with 44%). However, the absolute benefit of immediate ART was greater in the TEMPRANO trial (conducted in Cote d’Ivoire) than in the START trial (which was conducted in countries across the world), because the event rate in the control arms (mainly from TB and invasive bacterial infections) was higher in the TEMPRANO trial, reflecting the high co-infection risks that exist for individuals living with HIV infection in Africa, even with higher CD4 counts. No significant difference in mortality was observed between the study arms in either trial.^2,3^

There are two main concerns about ART initiation with CD4 counts > 500 cells/μL. Firstly, the risk of adverse events could outweigh clinical benefits. Secondly, adherence could be lower in asymptomatic patients. In both trials, immediate ART did not increase the risk of adverse events overall, nor did patients who started immediately have a higher risk of adherence problems, at least in the short term, as evidenced by the high proportion of patients achieving HIV viral suppression seen in the immediate arms in both trials (Table 1).

The HPTN052 trial^4^ previously demonstrated that ART prevented onward transmission of HIV within serodiscordant couples, which suggested that ART at HIV diagnosis for all may be an important strategy to help prevent the growth of the HIV epidemic at a public health level. However, such an approach would be difficult to justify if there were no individual benefit and potential individual harm. What these two trials have demonstrated is that there is indeed individual clinical benefit with no signal of harm during ~3 years of follow-up, providing significant additional support for the ‘test-and-treat’ strategy.

These were well-conducted randomised controlled trials with good participant retention and assured drug supply, and the vast majority of participants were on tenofovir/emtricitabine/efavirenz regimens in both trials. In less motivated patients, or in the context of inconsistent drug supply or different first-line ART regimens, these results should be treated with caution, as the modest benefits seen with early treatment may not be realised. In particular, if tenofovir (or abacavir) is unavailable and zidovudine or stavudine needs to be used in place, then the substantial side-effect profile of these drugs and effect on patients’ quality of life may outweigh the benefit of early ART.

## Isoniazid preventive therapy in patients on antiretroviral therapy

The TEMPRANO trial involved a separate randomisation of participants to 6 months of isoniazid preventive therapy (IPT) started one month after study entry, versus no IPT. The addition of IPT to ART provided added protection against active TB disease, even in these patients with relatively high CD4 counts. This finding was similar to those of a placebo-controlled trial conducted in Khayelitsha, South Africa, where 12 months of IPT prescribed to patients on ART reduced TB incidence by 37%.^5^ Our December 2014 guidelines^1^ recommended IPT for all patients on ART provided that there was no contra-indication to isoniazid, and active TB was not suspected based on symptom screen. Guidelines on duration of IPT based on tuberculin skin test results were provided. Given that southern Africa has the highest incidence rates of HIV-associated TB in the world, IPT as a TB-preventive strategy implemented within ART clinics should be prioritised.

## Updated recommendations of SA HIV Clinicians Society regarding antiretroviral therapy initiation

Our updated recommendations are shown in Box 1. It is important to note that these are the guidelines of the SA HIV Clinicians Society, and clinicians working within national public sector ART programmes should continue to follow the guidelines that pertain to their programme. Whilst National Departments of Health are very likely to consider a change in ART initiation policy in the near future based on these study findings, such a change will need to factor the financial cost, adequate planning of drug supply and health service capacity. Sustaining funding for ART programmes in resource-limited countries is a huge challenge currently.^6^

**Box 1:** Indications for antiretroviral therapy in adults with HIV infection.

**We recommend initiation of lifelong ART for all patients diagnosed with HIV infection**. The CD4 count and clinical stage of the patient should no longer be a consideration in the decision to start ART.
For patients who are asymptomatic with CD4 > 350 cells/μL, additional time (weeks to a few months) can be spent counselling and preparing the patient for lifelong ART with good adherence before starting. In those with CD4 < 350 cells/μL (and especially < 200 cells/μL), or with clinical indication for starting, there should not be undue delay.
Within ART programmes, it is important to factor in that the absolute benefit of ART is much greater at lower CD4 counts (there is a mortality benefit at CD4 < 350 cells/μL.^10†^ Therefore, planners and clinicians should prioritise and fast-track those with low CD4 counts (especially < 200 cells/μL); this is particularly relevant where there are ART shortages or anticipated stock-outs.

† Severe P, Juste MA, Ambroise A, et al. Early versus standard antiretroviral therapy for HIV-infected adults in Haiti. N Engl J Med. 2010;363:257–265. PMID: 20647201, http://dx.doi.org/10.1056/NEJMoa0910370

## Specific patient groups

For patients who are diagnosed with HIV during *acute seroconversion*, we continue to advise that those patients be counselled and initiated on ART. This should preferably be expedited ART initiation as there is evidence that this may limit the size of the HIV reservoir.^7^ However, we no longer recommend that these patients should have ART interrupted after 3 years. Rather, once ART is started in this situation, this should be lifelong ART, and this should be discussed with the patient. Additional counselling once the patient is established on ART may be required for patients who start ART in this acute situation because there is limited time for extensive counselling pre-ART and there is often considerable psychological distress around this time.

A minority of patients (< 1%) have very effective immune control of HIV infection and are able to control HIV viraemia at undetectable levels in the absence of ART – termed ‘*elite controllers*’. An argument could be made that such individuals do not require ART if their CD4 count is > 500 cells/μL. The START and TEMPRANO trials did not specifically address the question of whether early ART was beneficial in such patients, and it is highly unlikely that any sufficiently powered randomised controlled trial could ever be conducted focused on these individuals. In the absence of such data, we rely on indirect evidence. Firstly, elite controllers still have evidence of chronic immune activation and inflammation that may drive non-infectious morbidities.^8^ Secondly, elite controllers have been shown to have a higher rate of hospitalisation than patients who are virologically controlled by ART.^9^ For these reasons, we do advise starting ART in elite controllers too, with the same caveats regarding the patient being prepared. One important consideration in such patients is that careful attention should be paid to confirming the diagnosis of HIV before starting ART. They are typically patients who have a positive HIV ELISA test, undetectable HIV viral load, CD4 count in the normal range and are clinically well. The possibility of a false positive HIV ELISA test should be excluded either by performing a qualitative HIV DNA PCR assay or a Western blot assay. If the patient at some point previously had a detectable HIV viral load, this would also serve as confirmation. Such patients should be discussed with a laboratory virologist to assist with confirmation of HIV infection status.

In patients with *active TB* or *opportunistic infections*, the 2014 guidelines included guidance as to when to initiate ART in relation to treatment of the TB or opportunistic infection. This guidance remains unchanged.

We recommend ART initiation without delay in all *HIV-infected women who are pregnant or breastfeeding*, irrespective of CD4 cell count, to prevent mother-to-child transmission of HIV infection. We advise that such ART should be continued lifelong and not stopped after the pregnancy or weaning.

In *children and adolescents*, advice from the Society’s Child and Adolescent Guidelines Committee is that the ‘test-and-treat’ approach is supported in principle for all ages. However, there is an absence of data for the 5–15-year-old age group. Adherence issues for adolescents in particular and the lack of a simplified first-line regimen make ‘test-and-treat’ for 5–15– year-olds complicated. Please refer to national Department of Health guidelines for further advice regarding children and adolescents.
